# Complete Genome Sequence of Carbonic Anhydrase Producing *Psychrobacter* sp. SHUES1

**DOI:** 10.3389/fmicb.2016.01442

**Published:** 2016-09-13

**Authors:** Mengmeng Li, Xuejiao Zhu, Stephenson Wilkinson, Minsheng Huang, Varenyam Achal

**Affiliations:** ^1^Shanghai Key Lab for Urban Ecological Processes and Eco-Restoration, School of Ecological and Environmental Sciences, East China Normal UniversityShanghai, China; ^2^Department of Civil Engineering, University of WolverhamptonWolverhampton, UK

**Keywords:** whole genome sequencing, *Psychrobacter* sp., carbonic anhydrases, urease, biocement

Recent advances in biotechnology have allowed the study of new bacterial strains, which can produce enzymes that can be used in the bioremediation of heavy metals. Microbially induced carbonate precipitation (MICP) is a recent well-recognized process that has the potential to precipitate heavy metals, mainly those with a valency of +2 (Kumari et al., 2016). There are two enzymes, urease, and carbonic anhydrase, that play an important role in the MICP process. The role of carbonic anydrase (EC 4.2.1.1) in MICP is generally underestimated and most of the studies in past mainly focus on urease-producing microorganisms (Li et al., 2013, 2014; Kumari et al., 2014).

In the present study, *Psychrobacter* sp. SHUES1 was isolated from frozen alkaline soil sample collected at Shanghai, China. This bacterium produced lipase and protease at 4°C in a plate assay. The ability of *Psychrobacter* sp. to show extracellular lipolytic activity at low temperatures is widely known (Xuezheng et al., 2010); however, the remarkable property of this strain was in the precipitation of heavy metals including cadmium and zinc in parallel to the MICP process. Therefore, to know the type of enzyme or genes involved in the process of metal precipitation, this research aims to sequence the whole genome of *Psychrobacter* sp. SHUES1, and thus provide a genomic insight into its behavior.

Genomic DNA from *Psychrobacter* sp. SHUES1 was extracted using the DNeasy Blood & Tissue Kit (Qiagen, USA), and its quantity and quality were evaluated on the Qubit. The extracted DNA was subjected to whole-genome shotgun sequencing using the NEBNext Ultra DNA Library Prep Kit (Illumina, San Diego, CA). Library construction was performed with the following process: DNA fragmentation, end repair, adding “A” to the 3′ end, adaptor ligation and amplification. After library construction, the generated cluster was sequenced on an Illumina HiSeq2500 sequencing system, according to a paired end 2 × 125 nt multiplex program. 13,716,515 raw reads resulted in 13,144,818 quality-filtered trimmed reads, yielding a not less than 3 Mb genome size. *De novo* genome assembly was performed using SPAdes-3.5.0. After purification, the assembly produced 3,115,590 bp of sequence across 115 contigs with an N50 of 47,049 bp, with a longest sequence of 182,144 bp, and a G+C content of 43.5% (Table 1). Gene prediction and annotation were carried out using Prodigal_v2.6.1, blastp in the National Center for Biotechnology Information (NCBI) “nr” database. Gene ontology (GO) functional annotation of genes was carried out using the blast2GO algorithm, dominated by the following features: biological process (44%), molecular process (42%), and cellular component (14%). Clusters of Orthologous Groups (COG) annotation was carried out in the NCBI COG database using rpsblast. A total of 2627 protein-coding genes, 45 tRNA-coding genes, and 6 rRNA genes were predicted in the draft genome.

**Table 1:** **Genome features of *Psychrobacter* sp. SHUES1**.

**Attributes**	**Value**
Genome size (bp)	3,115,590
GC content (%)	43.57
Protein coding genes	2627
tRNA genes	45
rRNA genes	6
ncRNA genes	116
GenBank accession no.	LXWA00000000

The most significant finding of the whole genome sequencing of *Psychrobacter* sp. SHUES1 was the presence of carbonic anhydrase gene in it. Carbonic anhydrase participates in all physiological processes dealing with CO_2_ and HCO_3_, such as cellular pH regulation, calcification, acid, and ion transport (Smith and Ferry, 2000; Achal and Pan, 2011). It catalyses the interconversion of CO_2_ and HCO_3_, which ultimately promotes the precipitation of calcium carbonate in the presence of Ca^2+^ ions. Although there are a number of genome sequences of *Psychrobacter* sp. deposited in NCBI database, this is the first characterization of the genome sequence of strain SHUES1, which produces carbonic anhydrase which has a significant role in metal bioremediation based on the ability to promote the precipitation of metal carbonates. This sequencing result also suggests the importance of carbonic anhydrase in the MICP process which is a novel element in this field of research.

The present study is especially valuable in the area of biomineralization based on MICP processes, in the bioremediation of metals and in the development of microbial concrete (biocement). Urease is the main enzyme responsible in such studies; however, in our study the urease gene was not present in *Pyschrobacter* sp. SHUES1. This indicates the importance of carbonic anhydrase, as a less studied secondary enzyme for the MICP process. It is hoped that this research will encourage other researchers to look for this carbonic anhydrase precipitation pathway when carrying out MICP studies.

## Nucleotide sequence accession and culture collection number

The *Psychrobacter* sp. SHUES1 whole genome shotgun (WGS) project has been deposited at DDBJ/ENA/GenBank under the accession LXWA00000000. The version described in this paper is version LXWA01000000, and consists of sequences LXWA01000001-LXWA01000115. The detail information and data related to sequences LXWA01000001-LXWA01000115 can be accessed as well as downloaded at http://www.ncbi.nlm.nih.gov/Traces/wgs/wgsviewer.cgi?val=LXWA01&search=LXWA01000000&display=contigs. This strain has also been deposited in the China General Microbiological Culture Collection Center (CGMCC 1.15733).

## Author contributions

ML and XZ, performed experiments; MH and VA, analyzed data; SW and VA, wrote manuscript.

### Conflict of interest statement

The authors declare that the research was conducted in the absence of any commercial or financial relationships that could be construed as a potential conflict of interest.
